# Robot-aided therapy for upper limbs in patients with stroke-related lesions. Brief report of a clinical experience

**DOI:** 10.1186/1743-0003-8-18

**Published:** 2011-04-09

**Authors:** Federica Bovolenta, Patrizio Sale, Valentina Dall'Armi, Pierina Clerici, Marco Franceschini

**Affiliations:** 1 Medicine Rehabilitation NOCSAE Hospital AUSL of Modena, Modena, Italy; 2 IRCCS San Raffaele Pisana, Rome, Italy; 3 Clinical and Molecular Epidemiology, IRCCS San Raffaele Pisana, Rome, Italy

## Abstract

This study was aimed at verifying the improvement on the motor impairment and functionality in 19 patients with chronic hemiparesis after stroke treated with a robot-aided rehabilitation protocol using the ReoGo™ system (Motorika Medical Ltd, Israel), and at evaluating the persistence of the effects after 1 month. The study also focused on the actual possibility of administering the robot-aided therapy with the ReoGo™ for the upper limbs and on the patients' degree of acceptance and compliance with the treatment. Subjects underwent an assessment prior to the start of the rehabilitation project (T-1), one at the start (T0), one at the end of the treatment (T1) and one after one month from the end of the treatment (T2). The following tests were administered: (i) Fugl-Meyer (FM) upper limb; Ashworth scale (AS); Functional Independence Measure (FIM™) (T-1 - T2); (ii) strength evaluation; Visual Analogue Scale (VAS) for pain; Frenchay Arm test (FAT); Box and Block test (BBT); Timed Up and Go (TUG) test (T0 - T2). Additionally, the Euro-QoL questionnaire and a VAS for the treatment satisfaction were administered to the subjects. Non-statistical difference of scores at T-1 and T0 on almost the entire battery of tasks suggested a stable patients' performance prior to the start of the rehabilitation. With the exception of the Medical Research Council (MRC) and the AS sub-scales measuring -as appropriate- strength and spasticity of the shoulder, triceps and wrist, all scores showed a significant increase between T0 and T1. The improvement on the pain could not be proved significant (p = 0.10). A significant increase between T0 and T2 was found for all assessment scores, with the exception of the MRC for external shoulder rotators (p = 0.05) and of the AS for shoulder (p = 0.32) and wrist (p = 0.08). Substantial stability was observed between T1 and T2. Patients were capable of completing the treatment and showed good participant satisfaction. This pilot study led to the finding of a clinical improvement and excellent patients compliance. It is possible that the learning process experienced by the patients was robot-dependent, especially in consideration of the general maintenance of the achievements observed on all activities.

## 

Stroke is currently the most important cause of disability in industrialized countries; it is the main cause of functional impairment of the upper limbs, with important effects on participation to activities of daily living [1]. The upper limbs remain non-functional at 6 months post stroke in 30%-66% of cases, while only 5%-20% of the patients fully recover upper limbs functionality [2]. In the last 10 years rehabilitative therapeutic interventions have been developed to provide the best possible treatment both in acute and chronic phases. In this context, research showed that an efficient treatment must be intensive and specific [3], repetitive, functional and motivating for the individual [4,5] in order to allow for a continuous progression in the process of learning, acquisition and generalization [6,7]. The development of robot-aided tools for neurological rehabilitation is a very stimulating prospective when considering their highly rehabilitative potentials [8-10]. The objective of this study is to verify the improvement on the motor impairment and functionality after a robot-aided rehabilitation treatment with the ReoGo™ system and the persistence of the effects after 1 month. A focus will also be towards the actual possibility of administering the robot-aided therapy for the upper limbs with the ReoGo™ system and on the patients' degree of acceptance and compliance with the treatment. Subjects with stroke (chronic hemiparesis) and with the following inclusion criteria were prospectively recruited into this "before-after" study: (a) first acute event of cerebrovascular stroke; (b) unsuccessful conclusion of a previous rehabilitation program (with no evident improvement of motility recovery); (c) discontinuation from any upper limb rehabilitation treatment for at least 1 month prior to the first visit. The following subjects were not included in the study: (a) patients with severe cognitive, linguistic or perceptive impairment (Mini Mental State Examination (MMSE) < 24); (b) patients who refused consent to the study; (c) patients who interrupted the robotic treatment for more than 3 days. If the treatment was interrupted for less than 3 days, all missed sessions were recovered. All recruited patients signed an informed consent. Each patient underwent a treatment cycle using the ReoGo™ system. The treatment consisted of a total of 20 sessions lasting 45 minutes each, 5 days a week, for a total period of 4 weeks; the rehabilitative protocol designed by us consisted of exercises aimed at improving both movement type (i.e., the joints involved, with a proximal-distal progression) and mode of execution of the movement itself, with progression from passive movement to free movement. Forearm support was used during treatment. Specific tasks are described in Table S1, Additional file 1.

The first visit took place 1 month prior to the start of the treatment (T-1). Following visit were scheduled: immediately before the start of the treatment (T0), immediately after the end of the treatment period (T1), and after 1 month (T2) during which period patients underwent no specific rehabilitation for the upper limb. The assessment tasks were: Fugl-Meyer (FM) for upper limb with its subtest: Motor function, Sensation, Passive Joint Motion, Joint Pain. [Lindmark, Hamrin 1988) [11,12]; Strength evaluation of 10 muscles, according to the Medical Research Council (MRC) criteria [13]; Ashworth (AS) elbow, wrist and shoulder sub-scales for spasticity [14]; Visual Analogue Scale (VAS) for upper limb pain; Frenchay Arm test (FAT) [15]; Box and Block test (BBT) [16]; Functional Independence Measure (FIM™) motor sub-score [17,18]. In addition, subjects underwent a comprehensive evaluation using the Timed Up and Go (TUG) test [19]. Lastly, the Euro-QoL questionnaire for the quality of life [20,21] and a VAS for treatment satisfaction were also administered to the subjects. The evaluations timeline is detailed in table 1.

**Table 1:** Timeline of the evaluations performed on all patients during the study period

Tests	T-1	T0	T1	T2
**Fugl-Meyer motor function**	X	X	X	X
MRC*	-	X	X	X
**Ashworth Scale**	X	X	X	X
**Visual Analogue Scale *pain***	-	X	X	X
**Frenchay Arm Test**	-	X	X	X
**Box & Block Test**	-	X	X	X
**FIM™ *motor***	X	X	X	X
**Time Up and Go Test**	-	X	X	X
EURO-QoL*	-	X	-	X
**Visual Analogue Scale *satisfaction***	-	-	X	-

*MRC: strength evaluation of 10 muscles, according to the Medical

Research Council (MRC) criteria; EURO-QoL:

questionnaire on quality of life perception.

Specific aims of this study were: (i) to verify that subjects' performance was stable prior to the start of the robotic treatment. This was done by comparing the performance at T-1 and T0 with regards to the FM, FIM™ and AS; (ii) to detect the improvement on subject's clinical status and its maintenance at 1 month after the completion of the rehabilitation program. This was done by comparing the change in performance on all tests from T0 to T1 and T2 and from T1 to T2. The Wilcoxon test for paired data was applied to perform all time comparisons. The critical limit for significance was set at p < 0.05. The statistical software STATA/SE Release 10 was used to carry out all statistical evaluations.

Nineteen subjects were included in the study, 13 (68.42%) were males and 6 (31.58%) females; 7 individuals (36.84%) presented with left hemiparesis and 12 (63.16%) with right hemiparesis. The sample average age was 55.74 ± 12.60 years, with a range of 26-71; the average time elapsed since the acute event was 57.37 ± 92.37 months, with a range of 8-295 months. Table 2 summarizes descriptive, clinical and psychological sample information. The follow-up visit (T2) could not be carried out on 3 patients because of difficulties encountered by their relatives in reaching the hospital.

**Table 2:** Demographic, clinical and psychological sample characteristics

		**N**	**%**	**Mean ± Std.Dev**
**Time since Stroke**		19		57.37 ± 92.37
**Age**		19		55.74 ± 12.60
**Gender**	Males	13	68.42	
	Females	6	31.58	
**Affected Side**	Left	7	36.84	
	Right	12	63.16	
**Disease Severity**	Mild	9	47.37	
	Moderate	9	47.37	
	Severe	1	5.26	
**EURO-QOL**				
**MOB**	Yes	3	15.79	
	No	15	78.95	
	Unknown	1	5.26	
**CP**	Yes	10	52.63	
	No	7	36.84	
	Unknown	2	10.53	
**AU**	Yes	3	15.79	
	No	15	78.95	
	Unknown	1	5.26	
**DD**	Yes	4	21.05	
	No	14	73.69	
	Unknown	1	5.26	
**AD**	Yes	8	42.11	
	No	10	52.63	
	Unknown	1	5.26	

Stability in the patients' performance prior to the start of the rehabilitative treatment, supported by the non-statistical difference of scores at T-1 and T0 for all tasks, with the exclusion of the motor FIM™ (p = 0.01), was observed.

The improvement observed in the patients' performance from T0 to T1 reached statistical significance for the FM upper limb (p < 0.01) sub-scores, for the AS elbow sub-scale (p < 0.01), for the motor FIM™ (p < 0.01), for all muscles' strength according to the MRC criteria -with the exception of the external rotators of the shoulder (p = 0.18), triceps (p = 0.06), wrist flexors (p = 0.13) and extensors (p = 0.08)-, for the BBT (p < 0.01), for the TUG test (p = 0.01), and for the FAT (p < 0.01).

Similarly, statistical evidence for an improvement from T0 to T2 was found for the FM upper limb (p < 0.01), for the AS elbow sub-scale (p = 0.01), for the motor FIM™ (p < 0.01), for the VAS pain (p < 0.01), for all muscles' strength -with the exception of the external rotators of the shoulder (p = 0.05)-, for the BBT (p = 0.01), for the FAT (p < 0.01) and for the TUG test (p = 0.02). Statistical evidence in favor of a progressive improvement from T1 to T2 emerged for the motor FIM™ (p = 0.01) and the VAS (p = 0.02). The perception of the quality of life, as measured by the Euro-QoL, did not show statistically significant variations over time; the VAS for patients' treatment satisfaction had an average score of 98.68 ± 4.02. Table 3 summarizes the sample performance over time at all clinical tests.

**Table 3:** Performance at the clinical assessment tasks

		T-1 (N = 19)			T0(N = 19)			T1 (N = 19)			T2(N = 16)		
					
		Mean ± Std.Dev.	Median	Min; Max	Mean ± Std.Dev.	Median	Min; Max	Mean ± Std.Dev.	Median	Min; Max	Mean ± Std.Dev.	Median	Min; Max
**Fugl-Meyer Test (n = 18)**	***Upper Limb***	31.33 ± 17.42	33.5	5; 54	31.21 ± 16.92	33	7; 55	40.37 ± 18.57	49^b^	9; 62	41.75 ± 18.95	49.5^b^	9; 63
**Ashworth Scale (n = 18)**	***Shoulder***	0.67 ± 0.77	0.5	0;2	0.37 ± 0.6	0	0;2	0.16 ± 0.37	0	0; 1	0.25 ± 0.77	0	0;3
	***Elbow***	1.67 ± 0.91	1.5	0;3	1.79 ± 0.98	2	0;3	1.26 ± 0.93	1^b^	0;3	1.44 ± 1.03	1^b^	0;3
	***Wrist***	0.89 ± 1.02	1	0;4	1 ± 1	1	0;4	0.68 ± 0.67	1	0;2	0.63 ± 0.62	1	0;2
**FIM™ (n = 16)**	***Motor***	80.63 ± 16.22	82	53; 126	82.26 ± 13.88	83^a^	56; 126	85.21 ± 11.84	86^b^	69; 126	85.94 ± 6.32	88.5^b^,^c^	69; 91
**Visual Analogue Scale**	***Pain***				22.05 ± 26.33	15	0;80	11.58 ± 20.21	0	0;75	0 ± 0	_**0**_**b,c**	0;0
**Medical Research Council, muscles' strength criteria**	***Trapezius***				3.37 ± 0.76	3	2;5	3.79 ± 0.79	4^b^	3;5	3.94 ± 0.77	4^b^	3;5
	***Deltoid***				3.68 ± 0.58	4	2;4	4.37 ± 0.6	4^b^	3;5	4.56 ± 0.63	5^b^	3;5
	***Pectoralis Major***				3.74 ± 1.19	4	0;5	4.47 ± 0.77	5^b^	2;5	4.75 ± 0.45	5^b^	4;5
	***External Rotatores***				3.58 ± 1.12	4	0;5	4.11 ± 1.15	4^b^	0;5	4.25 ± 1.29	5^b^	0;5
	***Internal Rotatores***				32 ± 1.57	4	0;4	3.32 ± 1.57	4	0;5	3.56 ± 1.46	4	0;5
	***Biceps Brachii***				3.95 ± 0.97	4	2;5	4.53 ± 0.61	5^b^	3;5	4.81 ± 0.4	5^b^	4;5
	***Triceps Brachii***				3.74 ± 1.28	4	0;5	4.05 ± 1.08	4	1;5	4.31 ± 1.14	5^b^	1;5
	***Flexor Carpi***				3 4 0; 53	4	0;5	3.32 ± 1.67	4	0;5	3.63 ± 1.63	4^b^	0;5
	***Extensor Carpi***				2.84 ± 1.38	3	0;4	3.21 ± 1.51	4	0;5	3.5 ± 1.41	4^b^	0;5
	***Latissimus Dorsi***				2.74 ± 1.24	3	0;4	3.47 ± 1.12	4^b^	1;5	3.94 ± 1.06	4^b^	1;5
**Box & Block Test**					11.89 ± 11.69	12	0;38	16.95 ± 15.6	17^b^	0;45	17 ± 15.9	17^b^	0;54
**FrenchayArm Test**					2.47 ± 1.81	3	0;5	3.26 ± 2.05	5^b^	0;5	3.31 ± 1.96	4.5^b^	0;5
**Time Up and Go Test**					18.58 ± 7.9	17	10; 40	17.47 ± 8.55	14^b^	9; 38	16.25 ± 7.01	15^b^	8;34

The study showed a positive evolution of the limitation of activity and functionality for all subjects.

The sample had a baseline FM in line with other studies (Table 3) [10,22,23] and so was the increase in FM score (Lindmark and Hamrin) [10,22,23].

All subjects showed excellent compliance and remarkable satisfaction, highlighted by the results of the VAS rating and the absence of dropouts associated to intolerance to treatment. The increase in the motor FIM™ and the decrease on the VAS for the pain might be due to different strategies developed by the patients for compensating their motor deficits. This data is in accordance with Lauretani [2010] who observed a functional recovery after a rehabilitation treatment in patients discharged to home [24]. The statistically significant increase between T0 and T2 shows how the improvement observed immediately after completion of the rehabilitative protocol was maintained over time, even though the sample under examination included subjects in a stable disease stage. Our results are in accordance with those of Bosecker 2010, who studied 111 individuals with chronic impairment caused by stroke and trained with a robot [25]. Such a robot-guided treatment must be task-oriented, functional and motivating for the patient [4,5,23], and therefore capable of determining a process of learning, acquisition and generalization [6,7]. The use-dependent robot-aided instruments (intensive and repetitive treatment) may favor functional reorganization phenomena, typical of neuronal plasticity [3,26]. Our experience is also in line with studies [6,27] that confirm how this type of treatment does not negatively affect spasticity: in our sample, the elbow AS score was reduced, while no change, nor an increase in spasticity, was detected for the other joints [28]. The positive effect observed on the quality and speed of the walking performance, assessed through TUG both at T1 and T2, is also interesting. This data conforms with Esquinazi paper [29]. The results obtained from our study suggest that a motor and functional recovery takes place and can be interpreted as a possible result of the process of adaptation. In addition, it was also possible to observe a motor learning and generalization process, confirmed by the baseline improvements observed at T1 and maintained until 1 month after (T2), an indication of the fact that patients were not in a spontaneous recovery stage.

Further research with higher statistical power is necessary. The enrolment of a control group would provide a term of comparison for the identification of the time-dependent effects, thus addressing the question of whether improvements are therapy-dependent or effectively acquired. Eventual relationships between clinical outcome and potentially influential factors should be explored. Stronger evidence would be beneficial when coming to make the decision of using robotic devices as an integral part of the rehabilitation team activities, within a rehabilitation project designed accordingly to the specifications and objective requirements of each patient. In this context, subjects at different disease stages (i.e. patients in the acute and sub-acute phases) should be considered in future research. Indeed, while there are several studies with various robotic systems for the upper limb in acute/sub-acute stroke patients [30-32], only one study with ReoGo™ system in the sub-acute phase [33] has been carried out so far. The implementation of different protocols according to the severity of the impairment should also be considered.

The results obtained in terms of recovery in functionality and the restriction of participation, as well as in patients' compliance and operator satisfaction, are encouraging in spite of the limitations of this study. The significant improvements found from the baseline measurements to the end of the treatment may be an indication of a clinical-functional improvement, thus a presumed effectiveness of the REOGo™ instrument [26,34-36]. In conclusion, further research with neuro-imaging and/or TMS patterns, with an adequate control group, will be imperative to confirm these results.

## Competing interests

The authors declare that they have no competing interests.

## Authors' contributions

The overall design of the experiment was agreed upon by all authors. MF, PS and FB designed the overall study. FB, MF and PC defined the motor task. FB and PC selected the subjects and conducted all clinical evaluations. FB, PC and PS programmed the robot, including the Robot Training procedure, conducted all experiments and processed the data. VDA performed the statistical analysis. FB, PS, and VDA wrote the manuscript. All authors read and approved the manuscript.

## Supplementary Material

Additional file 1 **Reo Go Protocol**. The specific rehabilitation tasks. The assessment process is designed to view the patient's ability to perform specific exercises over time. The system is capable of measuring and displaying the patient's progress. The screen displays the activities of the patient on the machine, according to exercise dates. The following parameters can be changed: • **Number of repetitions **- how many times the exercise will be repeated • **Speed **- Values range between 10% and 200%. The 100% value is 5 degrees per second. • **Force **(the resistance force of the joystick) - 3 possible values - High, Medium, and Low. Low force will require less force from user to initiate movement. • **Motion mode **- Guided, Initiated, Step Initiated, Follow assist or Free • **Scaling **- Each exercise can be scaled according to patients' comfortable range of motion -i.e. stretched or squeezed from a center point. Values range from 0% to 200% of the original exercise. • **Random **- Each exercise can be run in Random mode, i.e. the computer selects the next point randomly from the points of the exercise. • **2D/3D mode **- for every exercise, the radius of motion may be fixed (2D motion) or changeable (3D motion). The system provides the following exercise operating methods: • **Guided mode **- the patient is actively assisted by the system. • **Initiated mode **- the patient initiates each trajectory segment (between two successive recorded points) by himself, overcoming a predefined force threshold and then is actively assisted by the system for the rest of the segment • **Step Initiated mode **- similar to Initiated, but each trajectory segment (between two successive recorded points), is further divided to predefined "sub-segments" (3 degrees each) to overcome force threshold. • **Follow Assist mode **- the handle moves at a slow speed towards the target. Once the user applies force to the handle in the specified direction the speed will be increased. **• Free mode **- the patient actively leads the movement by himself. A summary of total training time is also displayed. Pressing the individual dates will display a summary of training for the specific date. Click here for file
