# Supplementary material for: Robot-aided therapy for upper limbs in patients with stroke-related lesions. Brief report of a clinical experience
Source: J Neuroeng Rehabil. 2011 Apr 9;8:18. doi: 10.1186/1743-0003-8-18 (PMC3086823; doi:10.1186/1743-0003-8-18)
Supplement: Additional file 1 — Reo Go Protocol. The specific rehabilitation tasks. The assessment process is designed to view the patient's ability to perform specific exercises over time. The system is capable of measuring and displaying the patient's progress. The screen displays the activities of the patient on the machine, according to exercise dates. The following parameters can be changed: • Number of repetitions - how many times the exercise will be repeated • Speed - Values range between 10% and 200%. The 100% value is 5 degrees per second. • Force (the resistance force of the joystick) - 3 possible values - High, Medium, and Low. Low force will require less force from user to initiate movement. • Motion mode - Guided, Initiated, Step Initiated, Follow assist or Free • Scaling - Each exercise can be scaled according to patients' comfortable range of motion -i.e. stretched or squeezed from a center point. Values range from 0% to 200% of the original exercise. • Random - Each exercise can be run in Random mode, i.e. the computer selects the next point randomly from the points of the exercise. • 2D/3D mode - for every exercise, the radius of motion may be fixed (2D motion) or changeable (3D motion). The system provides the following exercise operating methods: • Guided mode - the patient is actively assisted by the system. • Initiated mode - the patient initiates each trajectory segment (between two successive recorded points) by himself, overcoming a predefined force threshold and then is actively assisted by the system for the rest of the segment • Step Initiated mode - similar to Initiated, but each trajectory segment (between two successive recorded points), is further divided to predefined "sub-segments" (3 degrees each) to overcome force threshold. • Follow Assist mode - the handle moves at a slow speed towards the target. Once the user applies force to the handle in the specified direction the speed will be increased. • Free mode - the patient actively leads the movement by himself. A [file 1743-0003-8-18-S1.DOC]

Table S1.

| 1° week: | FORWARD THRUST |
| --- | --- |
|  | HORIZONTAL ABDUCTION |
|  | FORWARD REACH 2D |
|  | HORIZONTAL REACH |
|  | FUNCTIONAL |
| 2° week | FORWARD THRUST |
|  | HORIZONTAL ABDUCTION |
|  | FORWARD REACH 3D |
|  | HORIZONTAL REACH |
|  | FUNCTIONAL |
| 3° week | FORWARD THRUST |
|  | HORIZONTAL ABDUCTION |
|  | FORWARD REACH 3D |
|  | HORIZONTAL REACH |
|  | FUNCTIONAL |
| 4° week | FORWARD THRUST |
|  | HORIZONTAL ABDUCTION |
|  | FORWARD REACH 3D |
|  | HORIZONTAL REACH |
|  | FUNCTIONAL |
